# A Perspective on Strategic Enrichment for Brain Development: Is This the Key to Animal Happiness?

**DOI:** 10.3389/fvets.2021.720422

**Published:** 2021-09-21

**Authors:** Dana L. M. Campbell, Caroline Lee

**Affiliations:** Agriculture and Food, Commonwealth Scientific and Industrial Research Organisation (CSIRO), Armidale, NSW, Australia

**Keywords:** emotional intelligence, cognition, neuro, welfare, affective state, behavior, livestock

## Abstract

Livestock animals are sentient beings with cognitive and emotional capacities and their brain development, similar to humans and other animal species, is affected by their surrounding environmental conditions. Current intensive production systems, through the restrictions of safely managing large numbers of animals, may not facilitate optimal neurological development which can contribute to negative affective states, abnormal behaviors, and reduce experiences of positive welfare states. Enrichment provision is likely necessary to enable animals to reach toward their neurological potential, optimizing their cognitive capacity and emotional intelligence, improving their ability to cope with stressors as well as experience positive affect. However, greater understanding of the neurological impacts of specific types of enrichment strategies is needed to ensure enrichment programs are effectively improving the individual's welfare. Enrichment programs during animal development that target key neurological pathways that may be most utilized by the individual within specific types of housing or management situations is proposed to result in the greatest positive impacts on animal welfare. Research within livestock animals is needed in this regard to ensure future deployment of enrichment for livestock animals is widespread and effective in enhancing their neurological capacities.

## Introduction

Livestock animals are sentient beings (1) with proven cognitive capacity and thus cognitive needs. These animals are capable of learning, self-control, self-awareness, show cognitive biases and have complex social relationships (2–5). They are also capable of experiencing emotions or affective states such as fear, anxiety, pleasure and potentially depression (6–9). Commercial livestock farming typically raises animals in less complex environments, when compared with what wild counterparts would experience. This reflects the logistics and economics of large-scale animal production, as well as consideration of injury, health, and disease risks that may come with increased environmental complexity. Thus, the animals may be healthy, but may not “have what they want” (10). These more controlled and simpler conditions can often result in undesirable behavioral patterns. High frequencies of abnormal behaviors such as stereotypies (11) and injurious inter-individual interactions [e.g., tail biting in pigs: (12); feather pecking in laying hens: (13)] indicate the environmental conditions may be suboptimal, contributing to welfare and production impacts. With the realization that sentient animals may not be best suited to less complex environments, an animal welfare focus over recent decades has driven a trend toward improving animal housing conditions to increase environmental and social complexity for enrichment purposes. Environmental complexity can be provided in many different forms to be enriching [i.e., physical, sensory, social, occupational, nutritional; (14)]. For this increased complexity to be termed “environmental enrichment,” the environmental modification must result in improved biological functioning of the captive animal (15). Enrichment when effective could include physical modifications to the environment through structural changes, cognitive challenges such as puzzles or learning tasks, social changes such as group vs. single housing, enhanced sensory stimulation such as music playback, or feeding variation that may stimulate foraging behavior. Commercially, some examples include the banning of conventional laying hen cages across several regions in favor of aviaries and furnished cages with valued resources such as perches and nest boxes (16); and the revision of farmed mink guidelines in Canada to include compulsory cage enrichment provision of manipulable objects (17).

While these increases in environmental complexity are a step in the right direction, they may not yet be enough to result in the desired animal welfare improvements in livestock species such as poultry, ruminants, pigs, and mustelids. In particular, while current enrichment interventions may help alleviate negative affective states by providing resources to meet an animal's behavioral needs, more may be required to encourage experience of positive affective states (8). Affective states are important in welfare assessment and have a direct relationship with affective neuroscience (8, 9). Thus, consideration of the neurological impacts of rearing (birth/hatching until maturity or slaughter age) and adult housing conditions (where animals may change housing environments once maturity is reached) must be made to ensure enrichment programs are achieving the desired positive impacts. Enrichment programs may need to be applied strategically to ensure an individual animal is best suited to the environmental conditions it will experience throughout its production life. This could include several different types of environments for animals that change housing across life stages (e.g., laying hens are reared in one facility and are moved to a different facility for the adult laying period). Enrichment is likely also critical for livestock in the prenatal period (i.e., pregnancy and incubation) and even prior [e.g., parent-stock impacts on subsequent chick development in laying hens: (18)] but the postnatal period will be the primary focus here. This perspective review explores the relationship between enrichment, developmental environments, neurological impacts, cognitive and emotional intelligence in livestock animals and the goal of positive welfare states drawing on evidence across humans and other animals where livestock research is limited.

### Defining Cognition and Emotion

Cognition is the term used for the mental thought processes of acquiring knowledge about the environment through learning, perception, and judgment as well as the manipulation and retrieval of this knowledge. Social cognition is the processing of information related to other individuals. Cognitive capacity is thus the ability that an individual or species has to carry out these actions, allowing understanding of the surrounding world. Emotions are the feelings that are experienced relative to specific situations and experiences. Emotional intelligence (EI) as first proposed by Salovey and Mayer (19), is defined as “the ability to monitor one's own and others' feelings and emotions, to discriminate among them and to use this information to guide one's thinking and actions” (p. 189). Emotional intelligence has been further divided into “ability EI” which measures an individual's theoretical understanding of emotions, compared with “trait EI” which measures how an individual may typically respond to an emotional situation (20). Multiple measures have been developed for assessing EI in humans, although it is still challenging to objectively quantify a comparatively abstract type of intelligence (21). While self-report assessment tools are not possible in animal research, performance in emotionally relevant situations allows measurement of EI in livestock. The specific term of EI is not frequently used in the animal-based literature but studies do indicate the presence of EI when compared with the descriptions in the originally proposed EI framework (19). For example, conditioned place preference/avoidance tests suggest livestock animals are able to avoid/seek previous negative or positive emotional experiences (22–24) indicating self-regulation of emotional states. Pigs show evidence of emotional contagion where naïve animals will recognize and respond according to the positive or negative emotional state of conspecifics (25, 26). There is also some evidence of empathetic behaviors across other livestock animals although the research is limited (27). Furthermore, individual variation in animal adaptation to new environments, appropriate vs. inappropriate (extreme) fear reactions to external stimuli, and performance in tasks that elicit conflicts in the animal in their focus (e.g., attention bias testing, or learning a new maze task) are all potential indicators of degrees of emotional intelligence.

Cognitive and emotional processes may be measured in different ways, but they are not distinct aspects of an individual's functioning, with increasing recognition and neurobiological understanding of their intertwine (28, 29). The interplay can be illustrated in processes such as emotional anxiety affecting attentional stimuli processing or disrupting working memory or chronic, unpredictable stress impairing behavioral flexibility in learning tests (30). Conversely, humans often use cognitive strategies to regulate their emotions such as through reappraisal of situations (28, 29). Thus, cognitive processing, emotions, and stress responses are intricately linked and impact an individual's functioning (28). Our understanding of the brain to date has predominantly come from research with humans and laboratory animals but there is the opportunity to both translate these findings to livestock for facilitating improvements in their housing environments, as well as increase our research specifically on livestock neural functioning.

### Impacts of Enrichments on Neural Development in Livestock

The brain, whether human or animal, begins its development *in utero*, and henceforth it can be impacted by its surrounding environment (31). Maturation of neural circuits occurs throughout life making an individual brain a product of its experiences (32, 33). In particular, the neonatal period (the weeks directly after birth) is one of rapid neuronal formation including critical periods in which external stimuli can have maximal effect (34). However, neurogenesis and maturation does also occur in the adult brain (35). As the brain matures it is modulated by the sensory input it receives including social contact such as maternal care [e.g., (36)], exercise [e.g., (37)], cognitive stimulation (38) and physical enrichments vs. impoverished surroundings (39). There is clear evidence from decades of research with humans, laboratory animals, and fish that provision of an enriched, stimulatory environment during both rearing and adult periods has positive impacts on the development, maturation, and plasticity of the brain [see reviews in (40–42)]. Given the enormous complexity of the brain, a full understanding of the pathways involved and precise modes of impact are still under investigation.

Within livestock animals the neurobiological studies of enrichment impacts are fewer. This is possibly due to relatively greater difficulty in analyzing brain function in larger livestock species compared with laboratory rodents as well as the comparative infancy of the application of neurobiological techniques within the field of animal welfare research. There is some evidence that beneficial brain-derived neurotrophic factor (measured in plasma as a proxy for brain concentrations) increases in pigs with exposure to foraging enrichment relative to barren housing (43). Enriched housing of pigs with straw for foraging has also resulted in increases in proteins that indicate greater capacity for both protein synthesis and neuronal activity (44). Changes within monoaminergic neurotransmitters suggested enriched pigs showed lower stress responses to the slaughterhouse experience than those housed in the barren conditions (44). Long-term cognitive enrichment resulted in gene expression differences in reward-sensitive opioid receptors in the amygdala of pigs suggesting positive states were being experienced (45). Pigs exposed to enrichments also had higher dopamine levels in their striatum relative to pigs from barren housing conditions (46). Chickens with more optimistic judgement bias responses following unpredictable stressors showed greater dopamine turnover in the mesencephalon (47). Additionally, more chicks from enriched conditions maintained an optimistic bias following the stressors relative to chicks from barren rearing environments (47). Comparatively the literature on neurological impacts of environmental enrichment in laboratory animals is much vaster but livestock results to date do broadly align with what has been previously demonstrated in other species [e.g., (40, 48–50)].

### Impact of Enriched Rearing Environments on Cognitive Capacity, Emotions, and Stress Responses in Livestock

Studies on the direct neurobiological impacts of enrichments in livestock animals are currently relatively limited. However, there is a large, and growing body of livestock research demonstrating enrichment impacts on indirect measures able to be taken on the whole living animal. These measures include cognition, affective states, judgement biases, and stress responses which behaviorally and physiologically support the underlying neurological changes that are likely occurring.

Physical enrichments and increased environmental complexity in chickens has been shown to reduce startle reactivity in young adults (51). Enrichment also improves chicks' responses to stress by buffering against more pessimistic judgement biases (47), reducing physiological stress and improving pro-inflammatory responses where increased swelling in response to an injected antigen is indicative of greater immunocompetence (52). Furthermore, enrichment during the rearing period will reduce fearfulness (53), and improve spatial cognition (54) in broilers and young adult hens, respectively, relative to those birds reared in more barren conditions which could have additional stress-reducing impacts. In calves, physical feeding enrichments can improve reversal learning and reduce reactive responses to novel stimuli (55). Both physical and cognitive enrichment in newly-weaned dwarf goats improve learning performance and exploration behavior (56). In pigs, environmental enrichments result in more optimistic judgement biases in gilts (57) and more rapid learning performance and better working memory in cognitive tasks in weaned piglets (58–60).

These (and other) studies highlight the positive cognitive and emotional impacts of providing enrichments across several livestock species, particularly during the developmental stages (i.e., before reaching sexual maturity or slaughter age for shorter-lived livestock animals such as meat birds, with the weeks directly after birth/hatching likely to be most critical). Furthermore, an enriched and more complex environment will reduce the occurrence of abnormal behaviors such as feather pecking in laying hens (61), locomotor stereotypies in mink (62), aggression and oral stereotypies in feedlot cattle (63) and oral stereotypies in dairy calves (64) which are all indicative of neural dysfunction (65). However, more research with livestock is needed to better understand the specific neural mechanisms of stereotypy development and how enrichment may exert its effect (66). Benefits are also not limited to just physical enrichments. For example, social enrichment in dairy cattle improved learning and performance in cognitive tasks (67) and there is growing interest in the provision of cognitive enrichment for livestock to facilitate mental engagement (68).

### Facilitating Animal Happiness

Given the compelling evidence of the neurological impacts of enrichment across species, coupled with the drive toward positive welfare for agricultural animals (8, 9), provision of enrichments should be a necessity moving forward to enable animals to fulfill their neurological potential. Cognitive capacity is improved through enrichment that facilitates proper development of an animal's natural cognitive abilities (69). As defined earlier (see section Defining Cognition and Emotion) cognition is also inextricably linked with emotions, stress or coping mechanisms and thus mental health. We see from human literature, children raised in depauperate conditions with poorer stimulation (e.g., orphanages, low socio-economic status) show reduced cognitive development (70, 71) that can be mediated by cognitive stimulatory intervention programs (72). Deficits in the developmental years can then impact functioning as adults with lower cognition in childhood linked to poorer mental health as adults (73, 74) such as an increased risk of developing anxiety later in life (75). Adult individuals with lower intelligence may be at increased risk for developing anxiety disorders following highly stressful events, hypothesized to be due to a reduced ability to cope with adverse circumstances (76). Furthermore, individuals with a lower intelligence quotient (IQ) also report being less happy than those with higher IQ (77). This can in part be mediated by socioeconomic status where greater access to resources may improve happiness scores (77), highlighting the reciprocity of the relationship between IQ, resource access, and happiness. There is also a clear, documented relationship in humans between happiness and emotional intelligence where people with higher emotional intelligence will report greater happiness scores, potentially through being able to better regulate negative emotions and capitalize on positive ones (78–80). Greater emotional regulation correlates with improved happiness and socioeconomic status (81). Emotional intelligence has even been demonstrated to compensate for low IQ (82) and can be a greater predictor of overall well-being than IQ (83).

Applying this evidence from human literature to livestock animals, individuals without sufficient early cognitive stimulation may be more likely to develop negative affective states or behavioral problems such as aggression. These individuals could develop poorer emotional intelligence which could increase their susceptibility to negative emotions and reduce their ability to experience positive states. They may also be less equipped to cope with stressful events such as moving between housing stages, or changes in social companions. They may have increased anxiety if their cognitive capabilities do not align with housing system expectations such as high system complexity (e.g., laying hen aviaries), or learning requirements of precision technologies such as automatic milking systems or virtual fencing (84). There could then be flow-on emotional impacts if the animals are unable to utilize system resources effectively. Evidence of such described impacts following more barren rearing environments have been documented in livestock as highlighted previously (see section Impact of Enriched Rearing Environments on Cognitive Capacity, Emotions, and Stress Responses in Livestock).

Enrichment may thus facilitate appropriate neural development for greater emotional intelligence and more experiences of positive states, as well as result in positive emotions through the process of engaging with the enrichment itself. Evidence from livestock animals to date demonstrates the innate curiosity drive to engage with novel stimulation in their environment. Young livestock animals will show motivation to explore and play with their physical environment (85–88). In humans, the degree of curiosity can also be highly correlated with the degree of emotional intelligence (89, 90), thus highlighting the potential interdependence between enrichment provision to stimulate neural development and future engagement with enrichments. Livestock will also actively engage with cognitive enrichments [e.g., pigs: (91); laying hens: (92); dwarf goats: (93)], and show motivation to learn (94) as well as physiological evidence of learning processes being rewarding (95–97). This evidence of a natural desire for enrichment engagement supports the negative impacts that the absence of such stimulation would have.

### Challenges of Environmental Enrichment and Future Strategic Enrichment Directions

While clear benefits of enrichment provision for livestock have been outlined, potential shortcomings of enrichment must be considered. By necessity, animals are in “unnatural” conditions where scientists, consumers, and stakeholders recognize that in many cases this can be sub-optimal for animal welfare. The challenge that researchers and livestock producers face, is the conundrum of trying to “fix” animals so that they have “natural” functioning, in ultimately what will likely always be “unnatural” conditions that place different demands on the animal than what their wild counterparts experience. For example, production animals may not have to select food, evade predators, find mates, or rear young, but do have to adapt to invasive husbandry practices, human interaction, reduced habitat choice, and reduced social choice.

Logistically, enrichment provision can be challenging under the constraints of commercial animal production and environmental control for proper health care. Enrichments may be able to be provided during rearing, but not in adult stages due to changes in housing environments, and/or management requirements. Or enrichments may be rapidly damaged/consumed resulting in limited opportunities for interactions. Removal of enrichment access has been shown to induce negative cognitive states relative to animals that never had enrichment access (57) and can decrease positive exploratory behaviors (98). Removal of enrichments may also induce inactivity which can be indicative of boredom (99, 100). High degrees of novelty in enrichment provision may have detrimental effects on neophobic individuals (101). Furthermore, high stimulation to promote extensive neural development could result in over-active brains that may actually have adverse impacts. In contrast with positive correlations between IQ and happiness in humans, some recent human-based evidence indicates that very high IQ (hyper brain) may increase the risk of mental and physical health problems such as anxiety and immune dysfunction (102). It is suggested that a hyper brain corresponds with hyper excitability of the body resulting in detrimental impacts (102). Intense enrichment provision may not extend the animal beyond their natural capacity as animal species will have neurological limitations, although we may not yet know precisely what these are (103). However, it is unknown whether intense enrichment would have similar “hyper brain” affective and physiological impacts in livestock animals or if these impacts are limited to the complexities of human nature and societal demands. Furthermore, intense enrichment within developmental periods may boost cognitive development to such a point that animals require more stimulation than can be provided by the environments they are subsequently housed in; potentially resulting in detrimental boredom states (104). Thus, a highly developed brain may suffer more in an only moderately stimulating environment, a hypothesis that warrants testing.

Consequently, strategic enrichment programs may provide a partial solution to both improved and positive animal welfare if they enable livestock animals to fulfill their neurological potential within their specific housing constraints, including adequately adapting to and coping with the production setting. Application of enrichments in terms of the type and timing to target key neurological developmental periods and pathways that may be most utilized with specific types of housing or management situations is likely to result in the greatest positive impacts on animal welfare. Targeted environmental and/or cognitive stimulation applied during rearing could optimize neurological development in the current conditions as well as best prepare the animals for their future housing system. By identifying the pertinent potential stressors the animals may encounter (e.g., frequent social change), or skills they may need (e.g., learning of technologies) enrichments targeted to develop the necessary specific brain areas or specific skills could enable animals to best adapt to their environment. This type of developmental strategy is being employed in some livestock management practices. For example, laying hens destined for aviary systems that require spatial navigation, are reared with ramps that facilitate the development of these skills (105). Different indoor rearing enrichment strategies will have contrasting impacts on how the adult hens utilize the outdoor area of a free-range system (106). Pigs reared with environmental enrichments as well as socialization show reduced stress and aggression following social remixing throughout life (107). However, there is great scope to better understand the neurological impacts that are occurring when animals experience specific types of enrichments (108) to optimize enrichment strategies further.

The notion of specific types of neurological development being better suited to specific types of environments has previously been suggested, where animals with differing hemispheric dominance may better cope with specific types of environments due to varying cognitive biases (109). Left hemispheric dominance is suggested to result in better coping with chronic stress situations (109), although ultimately the goal for improved livestock welfare is to avoid housing conditions that are likely to elicit chronic stress responses. Similar affective state frameworks have been presented for emotional lateralization and how this may impact animal reactivity and welfare (110). However, there is great scope for further research in this area to enhance the welfare of livestock species. Enrichments that are species relevant as well as housing environment relevant will likely have maximal benefit. Potential brain areas/skills of relevance could include cognitive flexibility. This flexibility is primarily through enhancement of the prefrontal cortex but can extend across other brain regions [reviewed in (111)] and could be important for animals that have to continuously adapt to changing environmental conditions such as free-range housing systems. Social enrichment may modify the hypothalamic-adrenal-pituitary axis and reduce subsequent emotional reactions to stress (112), thereby improving coping in animals that undergo stressful management procedures. Enhancement of the hippocampus may be beneficial to animals which require high spatial abilities in navigating their housing systems (113). However, these are simplified examples drawn from the extensive research conducted in laboratory animals. Further research with livestock accompanied by cross-disciplinary discussion with neuroscientists will maximize the benefits of enrichment strategies. Finally, further research on how enrichment during rearing conditions affects the brain of livestock animals will improve our understanding of more generalized benefits. Pair-housing in calves has been shown to improve adaptation during the weaning period (114) and cognitive performance in learning tests (67), indicating certain types of enrichment may translate across different areas of functioning to have multiple benefits to the animals.

In conclusion, this perspective highlights the importance of further research to understand the neurological impacts of different types of enrichments for livestock animals so we can ensure strategic enrichment programs are developed to best suit specific environments and experiences animals are likely to encounter. Enabling development of their cognitive and emotional intelligence should minimize negative affect and facilitate positive welfare states.

## Data Availability Statement

The original contributions presented in the study are included in the article/supplementary material, further inquiries can be directed to the corresponding author/s.

## Author Contributions

DC and CL conceived the ideas. DC wrote the first draft. CL provided feedback and both authors agreed to the final manuscript. Both authors contributed to the article and approved the submitted version.

## Funding

Open Access Publication fees were funded by the Commonwealth Scientific and Industrial Research Organisation (CSIRO) through a Julius Career Award to DC.

## Conflict of Interest

The authors declare that the research was conducted in the absence of any commercial or financial relationships that could be construed as a potential conflict of interest.

## Publisher's Note

All claims expressed in this article are solely those of the authors and do not necessarily represent those of their affiliated organizations, or those of the publisher, the editors and the reviewers. Any product that may be evaluated in this article, or claim that may be made by its manufacturer, is not guaranteed or endorsed by the publisher.
